# Prevalence of Bovine Brucellosis and Risk Factors Assessment in Cattle Herds in Jigawa State

**DOI:** 10.5402/2011/132897

**Published:** 2011-12-27

**Authors:** Farouk U. Mohammed, Salisu Ibrahim, Ikwe Ajogi, Bale J. O. Olaniyi

**Affiliations:** ^1^Animal Reproduction Unit, Jigawa Research Institute, Kazaure, Jigawa State, Nigeria; ^2^Department of Veterinary Surgery and Medicine, Faculty of Veterinary Medicine, Ahmadu Bello University, P.O. Box 720 Zaria, Kaduna state, Nigeria; ^3^Department of Veterinary Public Health and Preventive Medicine, Faculty of Veterinary Medicine, Ahmadu Bello University, P.O. Box 720 Zaria, Kaduna state, Nigeria; ^4^National Animal Production Research Institute Shika, Ahmadu Bello University, P.O. Box 720 Zaria, Kaduna state, Nigeria

## Abstract

A serological survey of Brucella antibodies was carried out in Jigawa State, northwestern Nigeria to determine the prevalence of the disease and risk factors among some pastoralist cattle herds. A total of 570 cattle of different ages and sexes selected from 20 herds across the four agroecological zones in the state were screened using Rose Bengal Plate test and competitive enzyme immunoassay. From the results 23 cattle (4.04%) were positive by Rose Bengal Plate Test while 22(3.86%) were positive with competitive enzyme immunoassay. The infection rate was higher in females than males. Cattle older than 3 years had a higher prevalence rate compared to age groups 2-3 years, 1-2 years, and less than 1 year. The prevalence rate was higher in cattle densely populated locations. Infection rate differs between herds with larger herds presenting high prevalence due to poor sanitary practice. It is hereby recommended that public enlightenment on adequate control and preventive measures using proper sanitary practice and calf hood vaccination are required.

## 1. Introduction

Brucellosis is a disease of major economic importance most especially in developing countries like Nigeria. Serological investigations and reports have demonstrated that brucellosis is endemic in Nigeria and evidence of infection has occurred in cattle, sheep, goats, camels, and human beings [1–7]. Traditionally pastoralists in Nigeria own more than 70% of the cattle population in the country [8] and they still practice the extensive system of husbandry where watering points and grazing areas are shared. Unfortunately there is uncontrolled movement of livestock within and into Nigeria, and the level of disease surveillance and quarantine is so low. This scenario poses threat of disease transmission. This study attempts to determine the prevalence and risk factors of brucellosis amongst some pastoralist herds in Jigawa State and also suggest appropriate control measures that can be applied for the state disease control and eradication program.

## 2. Materials and Methods

### 2.1. Study Area

**Figure 1:**
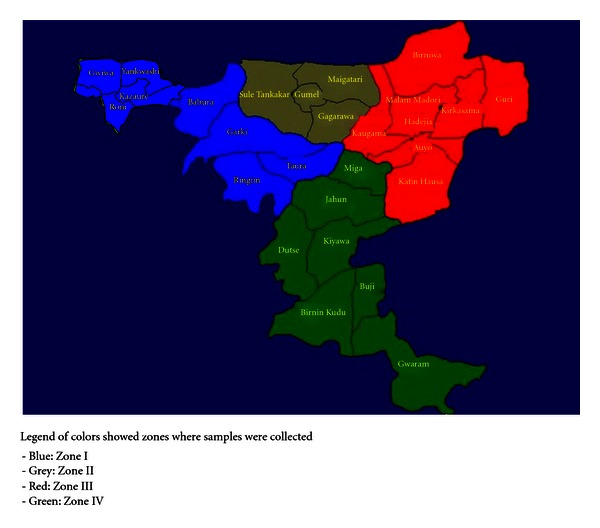
Map of Jigawa State.

Jigawa State lies between latitude 10°57 N and 13°03 N and longitude 8°08 E and 10°37 E and covers a total land area of about 22,410 sq.Km. The state has 27 local governments, and based on agroecological classification the state is divided into four zones. Zone I consists of Gwiwa, Yankwashi, Kazaure, Roni, Babura, Garki, Ringim, and Taura. Zone II consists of Sule tankarkar,Gumel, Maigatari, and Gaggarawa. Zone III consists of Hadejia, Birniwa, Mallam Maduri, Auyo, Kaugama, Kafin hausa, Guri, and Kirikasamma local governments while Zone IV consists of Birnin-kudu, Dutse, Gwaram, Kiyawa, Jahun, Miga, and Buji local government areas. The climate is semi-arid, characterized by a long dry season. The climatic variables vary considerably over the years and are erratic. The temperature is warm to hot. The mean annual temperature is about 25°C in the coolest month and 39°C the hottest month (Figure 1).

### 2.2. Sample Collection

A total of 570 serum samples were collected from cattle of different ages and sexes (mainly zebus of Rahaji, Bunaji, and Gudali) from 20 herds selected from the four agroecological zones in the state. They were screened for presence of *Brucella* antibodies by Rose Bengal plate test (RBPT) as described by Morgan [9] and Alton et al. [10] and further subjected to competitive ELISA (compelisa) test according to the manufacturer's instruction.

### 2.3. Serological Tests

The Rose Bengal plate test (RBPT) and Competitive Elisa (compelisa) were used in this study.

#### 2.3.1. Rose Bengal Plate Test

This was carried out using standard Rose Bengal plate test antigen obtained from Central Veterinary Laboratory, Weybridge, UK, according to the method of Alton et al. [10]. Equal volumes (0.03 mls) of antigen and test serum were mixed thoroughly on the glass plate of the test box using a toothpick, and the box was hand-rocked for four minutes.

**Control Setup**

The positive and negative controls were set up, and the results of the serology were compared. Any degree of agglutination was considered positive while absence of agglutination was regarded negative.

#### 2.3.2. Competitive Elisa (compelisa)

The competitive enzyme-linked Immunosorbent assay kit was obtained from Central Veterinary Laboratory, Weybridge, UK. The test was conducted according to manufacturer's instruction. Initially the diluting buffer, wash solution, stopping solution, conjugate solution, and controls were reconstituted as directed by the manufacturer. Test serum was added per each well of the microtiter plate which has sixty columns (wells).

100 *μ*L of the prepared conjugate solution was then dispensed in all wells. The plate was then shaken for 2 minutes in order to mix the serum with the conjugate solution. The plate was then covered with the lid and incubated at room temperature for 3 minutes. The content of the plate was then discarded and rinsed 5 times with washing solutions and then dried. 100 *μ*L of the substrate chromogen solution was added to all wells. The plate was kept at room temperature for 10 minutes. The reaction was slowed by adding 100 *μ*L of the stopping solution to each well.

**Control Setup**

20 mL of the negative controls was added to well A11, A12, B11, B12, C11, and C12, while another 20 mL of the positive control was added to wells F11, F12, G11, G12, H11, and H12. D11, D12, E11, and E12 serve as conjugated controls.

### 2.4. Interpretation

The results of the serology were compared with the control wells as follows: very weak or no color development in wells indicates negative result while a strong color development in wells indicates positive result.

## 3. Results

 The results of the screening test from Table 1 showed 23 samples positive by RBPT and 22 with CELISA. The prevalence rate for the two test were put at 23/570 (4.04%) and 22/570 (3.86%).

 Among the 200 males tested, 4 (2%) were positive with the Rose Bengal while 3 (1.5%) with the Competitive Elisa test. 19 out of the 370 females (5.1%) tested positive to both tests (Table 2).

 The prevalence among the age groups using Rose Bengal test showed that cattle older than 3 years had a prevalence of 13/233 (5.6%) followed by those between the ages of 2-3 years 5/91 (5.5%), 1-2 years 2/97 (2.1%), and below one year 3/149 (2.01%). The Competitive Elisa test showed similar results with the Rose Bengal test except among cattle older than 3 years where the Elisa reduced the prevalence to 12/233 (5.2%).

 The prevalence rate for the four agroecological zones varied from least 2.2% to 5.6%, However in zone III out of the 145 animals tested 7 were positive with RBPT while 6 animals were positive with the CELISA. Categorizing the herds showed that herds positive with RBPT test were confirmed by CELISA test, and the herd prevalence varied between 50% to 66.6%.

## 4. Discussion

The two tests showed degree of agreement; however the variation in prevalence by the two tests could be due to false positive. RBPT has been described as a highly sensitive but not specific test, while the CELISA is both a specific and sensitivity test and can eliminate cross-reaction due to heterogeneous bacteria and can minimize false positive.

 From the study the prevalence rate was higher in females than males as pastoralist usually keep female animals than males because of the offsprings and milk they get from them. Thus in all the herds sampled females are higher in number than males. However, Radostits et al. [11] have shown that erythritol, a polyhydric acid found in higher concentration in the placenta and foetal fluids of females than in seminal vesicles and testis of males can be responsible for females being more susceptible than males.

The prevalence was lower among the young animals screened in this study compared to the older ones. Usually young animals are protected by maternal immunity until when the immunity disappears, thus susceptibility seems to be low among them. The high prevalence seen in the older animals is demonstrating the chronic nature of brucellosis. The disease has been described as chronic and has been shown to increase with age, and most diseased animals carry the infection throughout their lives [11].

The difference in prevalence seen in the different locations is associated with the pastoralist husbandry practice that involves activities of cattle sharing grazing areas and drinking from the same points most especially in cattle densely populated areas like Zone I and Zone III. These areas have high cattle population because of the abundant pastures and grazing lands, thus allowing different animals to mix and possible transmission of the organism. A survey conducted in Kura local government area of Kano which is also a cattle concentration point by Bakari [12] discovered similar findings.

 The herd sizes were categorized into small, medium, large, and largest; the results showed increase in prevalence with greater herd size. From the study it showed that sanitary measures are poorer in larger herds compared to small herds. Unhygienic practices, cattle concentrations, and mixing encourage spread of the infection amongst the animals. A similar work done by Oloffs [13] and Fred et al. [14] in Uganda obtained the same findings.

In conclusion this study has indicated that there was significant association between the risk factors highlighted (age, sex, location, and herd size (Table 2)) and brucellosis *P* value < 0.05. All these factors play role in the epidemiology of the disease. It has also indicated that the CELISA, and RBPT tests can be used in brucellosis screening and eradication program. It is hereby recommended that public campaign on brucellosis using vaccination, improving sanitary practice and surveillance, is required for effective control and eradication. A wider survey should also be carried out in the state using serology, cultural isolation, and molecular characterization. The information that will be generated will help in obtaining a possible vaccine candidate.

## Figures and Tables

**Table 1:** Results of brucellosis screening using RBPT and CELISA.

TEST	RBPT negative	CELISA positive
RBPT positive	23	22
CELISA negative	0	1

**Table 2:** Prevalence of brucellosis by risk factors using RBPT and CELISA.

Variable	No. tested	RBPT (%)	ELISA (%)
Sex			
Males	200	4 (2)	3 (1.5)
Females	370	19 (5.1)	19 (5.1)
Age			
<1year	149	3 (2.01)	3 (2.01)
1-2 years	97	2 (2.1)	2 (2.1)
2-3 years	91	5 (5.5)	5 (5.5)
>3 years	233	13 (5.6)	12 (5.2)
Location			
Zone I	160	9 (5.6)	9 (5.6)
Zone II	130	4 (3.1)	4 (3.1)
Zone III	145	7 (4.8)	6 (4.1)
Zone IV	135	3 (2.2)	3 (2.2)
Herd size			
15–20 (small)	4	2 (50)	2 (50)
25–30 (medium)	6	3 (50)	3 (50)
35–40 (large)	7	4 (57.1)	4 (57.1)
45–50 (largest)	3	2 (66.6)	2 (66.6)
